# Do interprofessional education programs produce dissension that destroys them?

**DOI:** 10.3389/fphar.2015.00016

**Published:** 2015-02-06

**Authors:** Lon J. Van Winkle

**Affiliations:** Department of Biochemistry, Midwestern UniversityDowners Grove, IL, USA

**Keywords:** interprofessional education, interprofessional collaboration, dissension, multidisciplinary teams, conflict transformation, healthcare professions students

We have shown that healthcare professional students attitudes toward interprofessional collaboration improve in association with their work together on interdisciplinary teams in single courses (Van Winkle et al., 2012, 2013). Anecdotally, however, a few students complained that at least one team member from another healthcare professions program did not contribute their share to the success of the team. To test the possibility that these interventions to improve attitudes toward interprofessional collaboration might also have negative effects on some participants attitudes, we examined a new set of data more carefully for both positive and negative changes. Such a negative effect on students attitudes might also have a negative impact on establishment of interprofessional education programs.

Ninety five percent of first-year pharmacy students (202 of 213 students) and 100% (*n* = 96) of first-year prospective health professions students (defined in Van Winkle et al., 2013) completed the newly developed Jefferson Scale of Attitudes Toward Interprofessional Collaboration (JeffSATIC, Hojat et al., 2014) at orientation to their programs in August and September, respectively, 2014 (pre-course administrations of the scale). They then worked together in interdisciplinary learning teams to complete weekly biochemistry workshop exercises and regular discussions concerning the challenges and rewards of healthcare delivery. Eighty one percent of pharmacy students and 91% of prospective health professions students completed the scale again at the end of the 11-week fall academic quarter (post-course administration of the scale). Most students marked their survey forms with personal, four-digit ID codes so that their post-course survey scores could be matched to their pre-course scores for more powerful, paired statistical analyses. Statistical analyses were performed using GraphPad Prism 6 Software, Inc. (LaJolla, CA). This study was reviewed and found to fulfill the criteria for exemption by the Midwestern University Institutional Review Board.

Most students became more eager to collaborate after working in teams (*p* = 0.02, Effect Size (ES) = 0.21, paired *t*-test for first-year pharmacy students; *p* < 0.0001, ES = 0.55, paired *t*-test for prospective health professions students). However, the distributions of survey scores also broadened after post-course vs. pre-course administration of the scale to pharmacy and prospective healthcare professions students (*p* < 0.0001, *F*-tests to compare variances). The post-course distributions of scores appeared to be composed of at least two populations of students. Thus, the changes in some students survey scores were highly negative statistical outliers of the populations of score changes (Tukey, 1977; Ben-Gal, 2005). These sets of student outliers became much less eager to work with students from other programs (*p* = 0.0004; *ES* = 0.98, paired *t*-test for first-year pharmacy students; *p* = 0.004, *ES* = 0.95, paired *t*-test for prospective health professions students). While the latter groups of bona fide outliers comprised less than 8% of pharmacy and prospective health professions students in the study, the magnitudes of their negative changes in attitudes were relatively large. The average magnitudes of these negative changes exceeded the increases in mean scores among the remaining majority of students by more than six-fold. In our view, such highly negative experiences among a minority of healthcare professions students working on interdisciplinary teams could disproportionately influence administrators overseeing work toward establishing interprofessional education programs. Those with strongly negative feelings are likely to be much more vocal than students who favor such programs. The later social dynamic would undermine efforts to institutionalize existing pilot programs. In this regard, these students also worked on teams with other healthcare professions students in at least one other course during the same quarter term of 2014.

Thus, interprofessional education programs may themselves elicit dissension among participants about the desirability of such programs. While most participants in these programs become more eager to collaborate with members of other healthcare professions, some may become disenchanted with this collaboration. Such disenchantment might emerge, say, from negative experiences while working on teams with other healthcare professionals. The resultant greater dissension among participants could lead decision-making administrators to feel that the cost is not worth the benefit of supporting interprofessional education programs especially in the context of a healthcare education system that already seems to function adequately.

To counter such dissension, those of us who train healthcare professions students may have an obligation to help students examine changes in their feelings about collaboration especially if these changes are negative. Techniques, such as conflict transformation, likely are needed fully to resolve the complex social clashes among the cultures of various healthcare professions and their training programs. Conflict transformation has been used successfully to resolve and even to reconcile deeply-rooted, identity-based conflicts in the field of wildlife conservation (Madden and McQuinn, 2014).

The levels of conflict model of conflict transformation, discussed by Madden and McQuinn (2014), seems particularly appropriate to reconcile the identity-based differences that are likely to arise within interprofessional healthcare teams. Items on the JeffSATIC, that measure the factors “working relationship” and “accountability” (Hojat et al., 2014), include “All health professionals can contribute to decisions regarding the well-being of patients/clients” and the reverse-scored “The primary function of other health professionals is to follow, without question, orders by the physician who are treating the patients/clients.” Reconciliation of the deeply-rooted, identity-based feelings students may have toward the hierarchy implicit in these items is unlikely to be trivial but also unlikely to be thoroughly examined in most healthcare professions education programs. Instead, the faculty indicates to students how they should feel by promoting interprofessional healthcare professions training in the first place.

In the levels of conflict model, discussions should begin with a thorough and balanced discussion of the dissension and disagreement inherent in work by interprofessional healthcare students on the same team. What does each healthcare professional stand to lose through full and equal interprofessional collaboration? What will they each gain? How will patients/clients suffer because of such collaboration? How will patients/clients benefit from these efforts? Only through full resolution and reconciliation of these issues can the best vision be discovered and implemented for the long-term benefit of all concerned. Our bias is that this vision will include institutionalization of interprofessional education programs, but articulation of any vision should be the responsibility of all participants not just us.

## Conflict of interest statement

The author declares that the research was conducted in the absence of any commercial or financial relationships that could be construed as a potential conflict of interest.
